# Automatic generation and transfer of Yongju opera language using AIGC technology and style mapping

**DOI:** 10.1038/s41598-025-33142-z

**Published:** 2025-12-22

**Authors:** Xiaohong Meng, Ping Hu, Zhi Li

**Affiliations:** 1 School of Humanities and International Communication, Ningbo University of Technology, Ningbo, 315211 China; 2 School of Chinses Language and Literature, Hubei University, Wuhan, 430062 China; 3 School of Media and Law, NingboTech University, Ningbo, 315000 China

**Keywords:** Artificial intelligence generated content, Transformer-based conditional probabilistic generation, Conditional variational autoencoders, Automatic language generation, Language text style transfer, Computational science, Computer science, Information technology, Computational models, Data acquisition, Data processing

## Abstract

This research combines artificial intelligence generated content technology with style transfer methods to significantly improve the efficiency of generating and transmitting Yongju opera language content. To overcome fundamental challenges in traditional content creation, we first performed a comprehensive analysis of Yongju opera’s linguistic characteristics and established a multi-source dataset comprising 100 classical scripts, 50 contemporary scripts, and 500 lyric audio clips collected from the Ningbo Drama Research Institute and various opera platforms. The proposed framework features a dual-model architecture where the transformer-based conditional probabilistic generation (TFCPG) model acts as the content generator, while the conditional variational autoencoder (CVAE) functions as the style processor. The TFCPG transforms modern Chinese input into text complying with Yongju opera’s grammatical standards, and the CVAE enhances the text by incorporating dialect vocabulary and rhythmic patterns through style latent variables manipulation. Implemented in TensorFlow 1.4 with multi-task learning (batch size 32, Adam optimizer, learning rate 0.01), experimental results demonstrate that the TFCPG completes text generation in 33.32 min, representing a 56.6% reduction compared to the Transformer baseline. The model achieves a bilingual evaluation understudy (BLEU) score of 45.55 ± 1.32 (*p* < 0.01). In human evaluations, the system scored 4.35 for dialect authenticity, 4.18 for artistic expression, and 4.27 for cultural relevance. The CVAE component attained a BLEU score of 44.26 with 97.03% style transfer accuracy, exceeding the sequence to sequence (Seq2Seq) baseline by 10.28%. These comprehensive results confirm our approach’s effectiveness for Yongju opera language generation and style adaptation.

## Introduction

Under the impact of the wave of globalization and digitization, traditional culture is experiencing unprecedented challenges and changes. Yong Opera, a treasure of local opera originating in Ningbo, Zhejiang, and carrying a deep historical and cultural heritage, is also difficult to be alone. With its unique dialect charm, superb performance skills and far-reaching social connotation, Yong Opera occupies an important position in traditional Chinese culture. However, over time, Yong Opera has faced dilemmas such as declining audiences, shortage of creative resources, and aging audiences. The younger generation has gradually lost interest in Yong Opera, and its inheritance and development are in jeopardy. In this context, AIGC (artificial intelligence generated content) technology has brought new opportunities for the inheritance and innovation of traditional cultures such as Yong Opera. Relying on cutting-edge technologies such as natural language processing and computer vision, AIGC technology can automatically generate high-quality cultural content, and has demonstrated strong capabilities in many fields such as text creation, image generation, and music creation, greatly improving creative efficiency and enriching artistic expression forms. At the same time, AIGC technology has a strong learning ability. Through the learning and analysis of massive amounts of data, it can accurately grasp and imitate various artistic styles, which provides a solid technical support for the style migration and innovation of traditional culture.

This research focuses on the combination of AIGC technology and style mapping technology, aiming to realize the automatic generation and migration of Yongju language. This exploration has far-reaching significance for the inheritance and development of Yong Opera and even the entire traditional opera culture. As a precious heritage of local opera, Yong Opera contains irreplaceable cultural value in its unique artistic style and expression. With the help of AIGC technology, while retaining the essence of the language style of Yong Opera, it can inject new vitality and innovative forms of expression, thereby broadening the audience and promoting the vigorous development of Yong Opera in the new era. This is not only the empowerment of traditional culture by technology, but also the only way for traditional culture to be reborn in the context of the new era.

This research has made breakthroughs in the field of text generation and style transfer. The core contribution is the innovative integration of TFCPG and CVAE to build a new text style migration framework, which is significantly different from the existing methods. Traditional style transfer models rely on explicit style labels or simple encoder–decoder structures, which are difficult to capture the implied association between complex semantics and styles, and the generated text is prone to content distortion and style incoherent. In this study, with the help of TFCPG’s dynamic feature control mechanism, the semantic and stylistic characteristics of text are finely decoupled and reorganized. It can adaptively extract the core semantics of the input text, capture the potential distribution of the target style through the embedding space of the learnable style, and avoid the information loss of hard labels. The introduction of CVAE enhances the robustness of model generation, its variational inference optimizes the representation of hidden variables, and the conditional coding mechanism realizes the dynamic balance between style and content, so that the generated text not only maintains semantic integrity, but also naturally integrates into the target style. This integrated architecture breaks through the traditional limitations and cooperatively optimizes style and content under a unified framework for the first time. Experiments have shown that the model far exceeds the existing baseline model in terms of style intensity, content fidelity, language fluency and other indicators, and has unique advantages when dealing with long text and complex style conversion tasks.

## Related work

AIGC technology, which utilizes artificial intelligence to automatically generate diverse content, enables artificial intelligence (AI) to simulate human creation and automatically produce new content that follows specific rules through large-scale data training^1,2^. Recently, AIGC has received increasing attention and is growing exponentially. Shao Liangjing provided a comprehensive review of the current research status of AIGC in medicine, introducing the overview of AIGC and considering and summarizing the basic generative artificial intelligence models, tasks, target organs, datasets, and research contributions^3^. Intelligent services based on artificial intelligence are the core of the construction and practice of metaverse libraries. To this end, Zifan Cai introduced the development history and technological evolution of AIGC technology, focusing on the application scenarios of AIGC technology in smart library services^4^. Lv Yisheng believed that with the rapid development of artificial intelligence, cloud computing, and chip technology, AIGC is constantly evolving. From a cognitive and learning perspective, just as the human learning process goes from imitation to improvement, and then to creation, AIGC has also gone through the process of perceiving and combining the real world to predicting and improving the real world^5^.

With the rapid advancement of AI technology, language automatic generation technology has gradually integrated into various aspects of people’s daily lives. This technology utilizes deep learning algorithms to simulate human language habits and automatically generate smooth and logically rigorous text^6^. The exercises defined by language learning applications are teaching tools that apply new language concepts. Sebastian Gabriel proposed a complete model driven architecture method, which used universal testing languages for language conversion for model to model and model to text conversion^7^. Dong Chenhe comprehensively reviewed the research on natural language generation in the past 2 decades, especially the research related to deep learning methods for data to text generation and text to text generation, as well as new applications of natural language generation technology^8^. Metaphorical language generation refers to redefining a given text to include the desired word forms. Lai Huiyuan believed that it is a fundamental but challenging task in NLP, which has recently received increasing attention due to the promising performance brought by pre-trained language models^9^.

Language text style transfer is a sophisticated technique in NLP, which breaks the traditional limitations of textual expression, captures and analyzes different styles between texts, and then integrates these style features into new creations^10,11^. Text style transfer has a long history in the field of NLP. Jin Di conducted a systematic investigation into the study of neural text style transfer^12^. Text style transfer is one of the hot topics in the field of natural language processing in recent years, aiming to change the specific style or attributes of text through editing or generation while preserving its content. Chen Kejia reviewed existing technologies to advance research in this direction, with a focus on introducing unsupervised learning based text style transfer methods and further categorizing them into implicit and explicit methods^13^. Li Jin proposed applying the Conditional Generative Adversarial Network model to Mongolian font style transfer and provided relevant models, implementing corresponding algorithms and software to achieve the effect of font style transfer^14^. In recent years, the stylistic properties of texts have aroused the interest of researchers in computational linguistics. Researchers have begun studying the task of text style transfer, which aims to change the style properties of text. Hu Zhiqiang created a taxonomy to organize the text style transfer task model and provided a comprehensive summary of artistic states^15^. Using fixed vectors to represent a style is very inefficient, which results in weak representation ability of style vectors and limits the diversity of text with the same style. To solve this problem, Yang Haitong proposed a new neural generative model called adversarial separation network, which could jointly learn content and style vectors with strong representation ability and good interpretability^16^.

## Design and construction of language generation model for Yongju opera

### Language characteristics of Yongju opera

Yongju opera is based on the unique Ningbo dialect, incorporating rich dialect and ancient vocabulary, forming a strong local flavor. Its grammar is varied, often using inverted and omitted sentence structures, making the language appear compact and rhythmic^17^. Yongju opera uses straightforward and vivid language, with deep emotions that can finely depict characters and showcase the colorful aspects of life, bringing profound artistic feelings to the audience. The language of Yongju Opera is rooted in the Ningbo dialect, retaining numerous dialect vocabulary that, although rare in daily life, frequently appear in Yongju opera, thus adding a strong local flavor to the script. At the same time, Yongju opera also incorporates ancient and foreign words, which make the language more colorful and unique in flavor. Its grammar is also quite distinctive, retaining the differences between Ningbo dialect and Mandarin. Yongju opera makes use of the rich tones of Ningbo dialect, cleverly combining phonetics and arranging rhythms to make the lyrics sound rhythmic and easy to sing. The combination of diverse singing styles and language phonetics forms a unique musical style, and by controlling the pace, pauses, and stress, the language becomes more vivid, powerful, and engaging. The language characteristics of Yongju opera are shown in Table 1.

**Table 1:** Language characteristics of Yongju opera.

Feature category	Specific content	Example
Dialect vocabulary	Retain a large number of Ningbo dialect vocabulary, incorporate ancient words and loanwords	“Allah”, “sweet potato”
Grammatical structure	Use specific sentence patterns and word order such as inverted sentences and omitted sentences, and use rhetorical techniques such as overlap and repetition	“Walk around”, “take a look”
Phonological rhythm	The tone is rich and changeable, the singing is diverse, and the rhythm of the language is emphasized	Passionate and high-pitched plate cavity, soft and graceful curved card body
Expression	Vividly portray characters, show storylines, and convey thoughts and feelings	Humorous and sincere emotions
Artistic effect	Give the script a rich local color, enhance the audience’s emotional resonance and sense of regional identity	Unique musical style, fascinating language expression

### Data collection

Yongju opera, as a unique form of traditional Chinese opera in Ningbo, is widely loved by local and even national audiences due to its profound historical culture and unique dialect charm. However, with the development of the times, traditional Chinese opera faces challenges of inheritance and innovation. To protect and inherit the culture of Yongju opera and explore its modern new expressions, constructing a Yongju opera language generation model has become an important research topic. The primary task is to extensively collect data on the scripts and lyrics of Yongju opera, which is the foundation of model learning and determines the richness and accuracy of the generated content. It is necessary to collect classic scripts and manuscripts from libraries, archives, and opera research institutions, which provide rich language samples and reflect the historical and cultural significance of Yongju opera. In addition, attention should also be paid to modern new productions that incorporate more elements of the times and innovative thinking, bringing new styles to the model.

To systematically address data support and evaluation challenges in Yongyan opera language generation research, this study developed a comprehensive chain system covering data collection, enhancement, annotation, and evaluation. The benchmark dataset was constructed using 32 classic plays created by Ningbo Yueju opera Company between 1950 and 2020. It collected 12,800 multimodal data sets, generated 3840 variant data sets through phonological rules, and supplemented 2560 pseudo-parallel data sets using style transfer synthesis technology. This effectively resolved the scarcity of dialect data, ultimately forming a high-quality corpus validated by experts through two rounds of verification.

To generate 3840 phonological variation samples, this study developed a systematic phonological enhancement workflow integrating linguistic expertise with automated methods. The process comprises three key phases: First, phonological rule formulation. Linguistic experts from Ningbo dialect and Yongju opera research participated in analyzing classical scripts and recordings of Yongju operas, extracting characteristic phonological features such as initial consonant changes (voiceless vs voiced alternation), vowel substitution (“-ou” to “-ao” conversion), tonal patterns (level-oblique shifts), and syllable adjustments (erhuaization, reduplicated word usage). These features were systematically encoded into transformation templates to simulate dialectal phonetic variations. Second, rule execution. Using TFCPG as the input system, automatic partial substitution, insertion, or deletion operations were performed at sentence or phrase levels based on predefined phonological rules, generating variation samples consistent with Yongju vocal characteristics. Third, manual verification. The automatically generated samples underwent a second round of human review, where Yongju performers or dialect researchers conducted auditory evaluation and textual proofreading to eliminate invalid samples.

In order to construct pseudo-parallel data for training style migration models, this study relied on the CVAE framework to generate 2560 high-quality pseudo-parallel samples. The research uses the pre-trained CVAE model as the core converter. The model is pre-trained on a large number of Yong Opera texts and modern Chinese texts. Its encoder can map the input sentences to the decoupled content and style space, and the decoder can generate new sentences based on the target style code and content characterization. When generating, the same modern Chinese sentence is entered into the model, and its corresponding version in the Yongju dialect style is obtained by controlling the hidden variables of the style, forming a pseudo-parallel pair.

These pseudo-parallel pairs are approximate matches rather than exact matches. Due to the inherent differences in expression between dialects and modern Chinese, complete word-by-word correspondence is neither feasible nor in line with the laws of art. In order to ensure parallelism and semantic consistency, a number of mechanisms are adopted: the content encoder only captures semantic information that has nothing to do with style and retains the content of the original sentence proposition; a content retention loss function based on cosine similarity is used to constrain the similarity between the generated text and the original text in the semantic space during training; all sentence pairs are evaluated by two-way semantic matching, and the similarity is higher than the threshold value; the generated pseudo-parallel data has also been double-reviewed by Yongju experts to ensure that it conforms to dialect habits and has consistent semantics, which provides a reliable basis for style migration model training.

Ningbo dialect is the foundation of Yongju opera, so the organization of dialect vocabulary is also crucial to provide rich authentic vocabulary for the model and make the generated text more vivid. The statistical information of each dataset in Yongju opera language is shown in Table 2.

**Table 2:** Statistical information of various datasets in Yongju opera language.

Dataset name	Data source	Data type	Amount of data
Collection of Classic Scripts of Yongju Opera	Ancient Books Department and Archives of Ningbo Library	Full script	100 units
Collection of Modern Plays of Yongju Opera	Ningbo Institute of Opera and Opera, Network Resources	Full script	50 units
Yongju Opera Lyrics Audio Library	Opera website, personal collection, theater archives	Audio file	500 segments
Yongju Dialect Vocabulary Library	Dialect dictionary, field collection	Vocabulary list	5000 words
Summary and Introduction of Yongju Opera Script	Public publications and online materials	Text abstract	200 articles
The author of the Yongju Opera script and the background of the creation	Opera history books, interview records	Text information	100 pieces

As shown in Table 2, a Yongju opera lyric audio library containing 500 precious audio clips is successfully constructed through various channels such as opera websites and theater archives. These audios intuitively demonstrate the unique phonetic charm of Ningbo dialect. To enrich vocabulary resources, dialect dictionaries and field collected Yongju Opera dialect vocabulary are also integrated, with a total vocabulary of over 5000 words, fully ensuring the authenticity and comprehensiveness of the vocabulary. These detailed data have laid a solid foundation for constructing the Yongju opera language model, helping to generate more authentic and vivid Yongju opera language texts.

### Data preprocessing

In order to improve the efficiency of Yongju language generation and transmission, it is necessary to build an efficient data collection and preprocessing framework to ensure that the model can generate high-quality content within a reasonable time. Extensive collection of opera script and lyrics data from libraries, archives and opera research institutions, etc., after careful preprocessing, the quality and richness of the data are guaranteed. The TextRank algorithm is used to extract keywords and use them as binding information in the generation, which improves the relevance and accuracy of the generated text. The multi-task learning strategy is adopted to enable the model to achieve smoother and more natural language expression while maintaining a high accuracy rate, and the overall efficiency can be improved. In view of the problem of low similarity between language conversion and style, a style mapping network based on CVAE is introduced. In-depth analysis of key elements such as phonology, sentence pattern, vocabulary selection, and emotional color of Yongqu text data, quantify them into style feature vectors, and after a large number of Yongqu text learning and training, generate feature vector sets that match various styles to provide strong support for style conversion. In order to achieve the goal of combining AIGC with style mapping technology to obtain real output, TFCPG is responsible for generating high-quality drama language text, and CVAE focuses on style conversion. In order to verify the effectiveness of the framework, a detailed ablation study was carried out to understand the unique value of each component and its impact on the final result by removing or modifying some components of the model. For quantitative evaluation, not only report the evaluation indicators, but also calculate the standard deviation and confidence interval, clarify the sample size and selection basis, and fine-tune all baseline models in the same data set to ensure fair and scientific comparison.

#### Keyword extraction

The keyword extraction task aims to automatically extract core vocabulary from articles or sentences, which highly summarize the content of the original text^18,19^. For the text of Yongju opera, it is expected to extract keywords through unsupervised methods as constraint information in the subsequent generation model. Simple frequency-based extraction methods are not conducive to low-frequency words. Although the term frequency-inverse document frequency algorithm can enhance the importance of low-frequency words, the effect is often not ideal^20,21^. Therefore, this article adopts the TextRank keyword extraction algorithm^22,23^. This is a sorting model based on graph structure that can automatically extract keywords from documents. Specifically, the algorithm first uses a graph structure to represent the Yongju opera script, where nodes represent vocabulary and edges represent co-occurrence relationships between vocabulary. After the composition is completed, the importance of each word is determined through iterative calculation of node weights, and the importance ranking of keywords in the Yongju opera text is finally obtained. The iterative calculation formula for node weights is expressed as:

1 $$RT\left({W}_{i}\right)=\left(1-d\right)+d*\sum_{{W}_{j}\in ln\left({W}_{i}\right)}\frac{{r}_{ji}}{\sum_{{W}_{a}\in out\left({W}_{j}\right)}{r}_{ja}}RT\left({W}_{j}\right)$$

In Eq. (1), $ln\left({W}_{i}\right)$ and $out\left({W}_{j}\right)$ respectively represent the set of adjacent points with node ${W}_{i}$ as the entrance and exit; ${r}_{ji}$ represents the weight of the directed edge from node ${W}_{j}$ to node ${W}_{i}$; $d$ is a positive number less than 1. In Yongju opera text, sorting can be done based on the weight $RT$ of words, and the largest word can be selected as the keyword.

#### Participle

Before processing Yongju Opera text to establish a language text generation model, a series of detailed text processing steps must be carried out. The primary task is to identify and process punctuation marks and non-Chinese characters in the text corpus. Usually, punctuation marks are considered as part of stop words and need to be removed from the corpus to eliminate noisy information. In addition, the corpus often contains redundant words, such as Arabic numerals, which need to be converted into Chinese expressions. This process is called corpus regularization. After normalization is completed, the sentence enters the segmentation stage and is segmented based on different punctuation marks^24^. At the same time, based on the original method, in order to further optimize the segmentation results, this study applies the reverse matching algorithm, aiming to achieve error detection and correction in the segmentation process. The basic idea is to reverse the text sequence to be processed after completing forward matching, and then perform reverse matching segmentation. This method can significantly improve the accuracy of word segmentation and reduce error rates.

#### Denoising data

When cleaning the audio data of Yongju opera lyrics, a series of tedious and detailed tasks are faced, which are crucial for improving data quality and laying a solid foundation for subsequent analysis, processing, and application. The vocal audio of Yongju opera is accompanied by various types of noise and redundant information. Therefore, the primary task is to collect high-quality live performances or professionally recorded audio from multiple channels to ensure the reliability of the data. Next, the professional audio software is used to perform preliminary denoising on the collected data. During this process, high-pass filter technology plays a crucial role. High-Pass Filter (HPF) is mainly used to filter out low-frequency components in audio, which often contain unnecessary background noise and environmental noise^25,26^. Given that the main frequency spectrum of Yongju opera’s singing and speech is concentrated in the mid to high frequency range, the high-pass filter can more effectively remove low-frequency noise, significantly improving the clarity and recognition of lyrics.

The transfer function of a high-pass filter (HPF) can be expressed as:

2 $$H\left(g\right)=\frac{1}{\sqrt{{g}^{2}+{g}_{a}^{2}}}$$

**Fig. 1:**
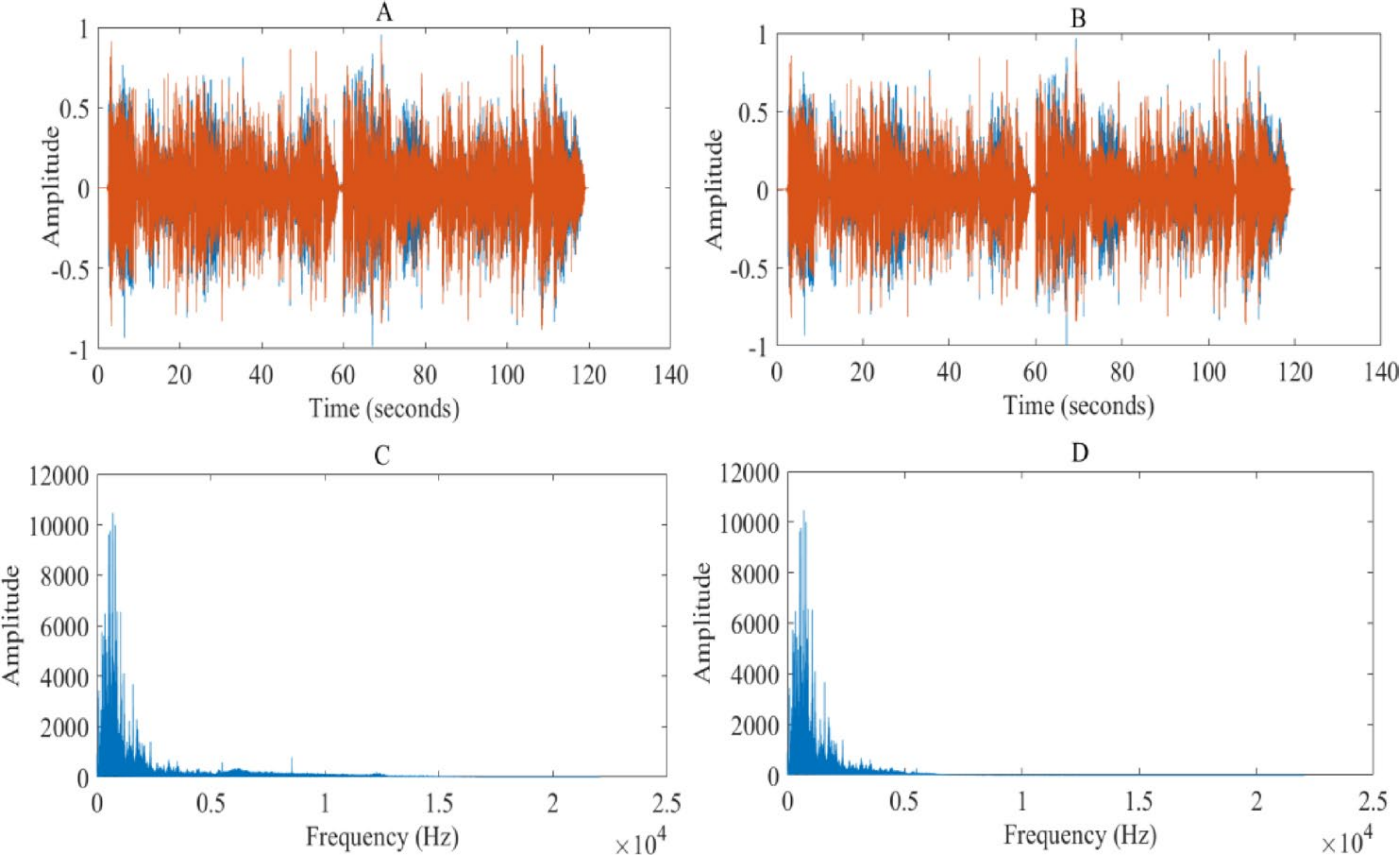
Noise reduction and cleaning effect of Yongju Opera lyrics audio data based on high-pass filter.

In Eq. (2), g is the frequency of the input signal; ${\mathrm{g}}_{\mathrm{a}}$ is the cutoff frequency of the high-pass filter, and signals below this frequency are attenuate; $\mathrm{H}\left(\mathrm{g}\right)$ is the filtered signal response, indicating that frequency components above the cutoff frequency are retained. The denoising and cleaning effect of Yongju Opera lyrics audio data based on high-pass filter is shown in Fig. 1.

As shown in Fig. 1, A–D represent the time-domain waveform of the original audio, the time-domain waveform of the filtered audio, the frequency spectrum of the original audio, and the frequency spectrum of the filtered audio, respectively. The high-pass filter has significant advantages in audio noise reduction. This filtering method has extremely strong selectivity and significantly improves the signal-to-noise ratio of audio signals, making the processed audio clearer and purer. Compared to the original audio, the denoised audio effectively reduces low-frequency noise such as background buzzing and low-frequency noise, while highlighting high-frequency speech and music components, providing listeners with a more comfortable auditory experience.

### Model construction

After the data collection and preprocessing work is completed, the next focus is to use AIGC technology to train and optimize the Yongju opera language generation model. The core of this step lies in selecting the appropriate model structure, developing scientific training strategies, and improving model performance through continuous optimization. Considering the complexity and artistic characteristics of the Yongju opera language, sequence generation models from deep learning are adopted, which have demonstrated excellent performance in the NLP field and are capable of handling advanced tasks such as long text generation and context understanding. Therefore, a transformer-based conditional probabilistic generation (TFCPG) model is chosen for automatic generation of Yongju Opera language text. TFCPG has a rich structure and is very suitable for processing language generation tasks. From the perspective of information conversion, language generation can be divided into two types: open and non-open. The TFCPG model constructed in this article is responsible for receiving input data at the input layer, which includes diverse information such as the theme number, keywords, and script audio of Yongju opera text. The word embedding layer is responsible for converting the input text information into vector representations in a high-dimensional semantic space, thereby helping the language model to better understand the text content. Finally, the output layer selects the most suitable text as the next part of the Yongju opera text based on the processing results of the multi-head attention layer, ensuring that the generated text is both coherent and in line with the characteristics of Yongju opera.

**Fig. 2:**
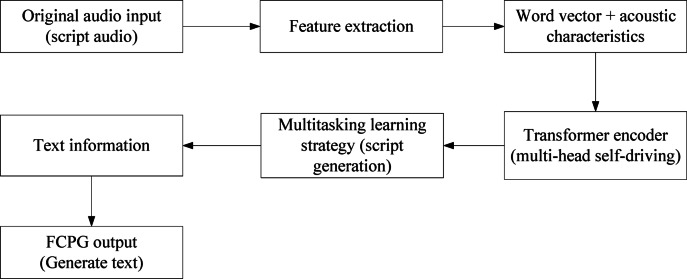
Schematic diagram of the whole process of automatic language generation and style migration of Yongju language.

In order to improve the clarity of the input description of the TFCPG model, it is necessary to explain in detail the method and technical details of the script audio, text, classification information, and acoustic data encoding integrated into the model. The script audio is the original audio file of the Yong opera lyrics, which has unique dialect phonological characteristics. Before entering the model, voice recognition technology is used to convert the audio into text format; acoustic characteristics such as Mayer frequency cepstral coefficients (MFCCs) are extracted as supplementary information. For text data, word segmentation technology is used and the vocabulary constructed based on the Yongju dialect is processed, and then converted into a word vector representation. After the text and acoustic features are embedded in their respective layers, they are cross-fused in the Transformer encoder part, allowing the model to capture the complex relationship between text and sound. In order to better show the whole process from input to output, a flow chart shown in Fig. 2 is designed.

When the TFCPG model processes multi-modal (text, audio, image) and four types of text data (complete script, vocabulary, text summary, text information), it uses multi-modal feature fusion and cross-modal alignment mechanisms to achieve collaborative optimization. For text data, the complete script is used as long text input, and the contextual semantic features are extracted by a hierarchical encoder, and the key plot information of the text summary is used to guide attention and strengthen the narrative logic of the generated text; the vocabulary data is aligned by the dialect dictionary and turned into an embedded vector containing phonology and semantics, which interacts with the complete script features at the attention level to supplement dialect-specific expression. In terms of audio data, it is first converted into text by speech recognition, and then the pre-trained Wav2Vec2.0 model is used to extract super-segment characteristics such as tone and rhythm. After the projection layer is mapped to the text feature space, multi-head attention calculation is performed with the semantic features to generate text rhythm matching and drama singing.

In order to capture the unique style and charm of Yongju opera language more accurately and deeply, it is particularly important to combine multi-task learning (MTL) with TFCPG model. MTL optimizes model performance by simultaneously processing multiple related tasks, which share the same input data but have different output objectives^27,28^. When building the Yongju opera language model, it is possible to carefully design shared layers and task specific layers to achieve effective MTL. When implementing MTL in the TFCPG model of Yongju Opera language, the first step is to build a reasonable model architecture. Unlike traditional autoregressive language models, the MTL strategy adopted in this article integrates a bidirectional encoder-decoder structure and utilizes both autoregressive and autoencoder methods for pre-training. Among them, the encoder part draws on the bidirectional feature representation autoencoder structure of the BERT (bidirectional encoder representation from transformers) model, while the decoder part adopts the autoregressive structure of unidirectional feature representation^29^. In the pre-training stage, the encoder receives text corrupted by any noise function as input, while the decoder restores and reconstructs these corrupted texts using one-way feature representation autoregression. In order to ensure that the generated text meets the format and rhythm requirements of Yongju Opera, the format and rhythm information of ${I}_{i}^{1}$ are used as inputs for the multi-head format and rhythm attention block. The specific calculation formulas are as follows:

3 $${F}_{i}^{1}=MN\left(FFN\left(\widehat{{F}_{i}^{1}}\right)+\widehat{{F}_{i}^{1}}\right)$$

4 $$\widehat{{F}_{i}^{1}}=MN\left(IK-ATT\left({P}_{i}^{2},{K}_{\le i}^{2},{W}_{\le i}^{2}\right)+{F}_{i}^{1}\right)$$

In Eqs. (3) and (4), $IK-ATT$ is the format and prosody attention layer, and the format and prosody information ${P}_{i}^{2}$ are used as key value vectors to obtain the output vector of this layer. After being overlaid by M layers, the output vector $MN$ is obtained. For different tasks, specialized decoders are designed to process the information provided by the shared encoder and generate outputs that meet the requirements of the task. For example, the lyric generation task is equipped with a sequence generation model that can generate melodious and semantically coherent lyrics; the plot prediction task uses a prediction model that is good at capturing the development of the story; the role dialogue task is achieved through a dialogue generation model that can simulate different character language styles and dialogue logic. The design of these task specific decoders fully considers the characteristics and requirements of each task to ensure that the generated content not only follows the artistic standards of Yongju opera, but also has high practicality and innovation.

In this study, the MTL strategy is used to improve the performance of the TFCPG model in the language generation of Yong opera. It can cover different dimensions of the script generation of Yong opera, and ensure that the generated content has both high-quality language expression and the unique artistic style and cultural connotation of Yong opera. Content prediction is the core task of MTL. With the help of the Transformer architecture, the model can capture the complex dependencies of long text sequences and generate coherent and contextual lines of Yong Opera. While ensuring smooth language and reasonable logic, it accurately presents the unique dialect vocabulary, grammatical structure and phonological rhythm of Yong Opera. Style classification is a key task. In view of the unique dialect charm and performance form of Yong Opera, it helps the model identify and transform the style characteristics of the text, and transform other style languages into dialect characteristic lines that conform to the artistic norms of Yong Opera. Auxiliary tasks such as sentiment analysis and character dialogue simulation are also essential. Sentiment analysis enables the model to understand and express specific emotional colors, enhancing the artistic appeal of the text; character dialogue simulation generates dialogues that fit the character and background of specific characters, making the script more vivid and realistic.

During training, balancing the loss functions of different tasks is the focus. Using weighted summation to integrate learning goals, each task has a corresponding loss function, such as the cross-entropy loss function for content prediction, the classification loss function for style classification, and then the weight is given according to the importance and contribution of the task to form a total loss function, in order to optimize the model training. The formula is as follows:

5 $${L}_{total}={\omega }_{1}*{L}_{content}+{\omega }_{2}*{L}_{style}+{\omega }_{3}*{L}_{auxiliary}$$

In Eq. (5), ${L}_{content}$, ${L}_{style}$ and ${L}_{auxiliary}$ represent the loss functions of content prediction, style classification, and auxiliary language goals, respectively. ${\omega }_{1}$, ${\omega }_{2}$ and ${\omega }_{3}$ are the weight coefficients of the corresponding tasks. These weight coefficients can be determined by performance tuning on the verification set to ensure that the entire model can achieve the best overall performance on various tasks.

The feature system design of the automatic generation and migration system of Yongqu language closely fits the uniqueness of Yongqu language and the requirements of the generation task. The system integrates three types of data sources: text, audio, and image, and extracts the five core feature categories of dialect vocabulary, sentence structure, phonological rhythm, emotional expression, and style migration. Dialect vocabulary characteristics are derived from dialect dictionary collation and script annotation, including Ningbo dialect-specific and ancient vocabulary, which can strengthen the local cultural attributes of the generated text; sentence structure characteristics extract special sentence patterns from classic scripts through grammatical analysis tools, combined with the key plot of the text summary, to ensure that the generated text conforms to the narrative rhythm of the Ningbo opera; phonological rhythm characteristics use speech recognition technology to convert the audio of the lyrics, extract the parameters such as tone changes, and combine the pure audio signal after noise reduction to guide the rhythmic design of the text; emotional expression characteristics With the help of character dialogue analysis and voice emotion recognition technology, extract emotional color parameters from the text summary and audio to adapt to the emotional needs of different performance scenes; style migration The features are based on the CVAE model to learn recessive features from the style corpus, and the adaptive conversion of modern text to classical drama style is realized through conditional variational inference. During model training, TFCPG uses a multi-head attention mechanism to integrate text and auxiliary features, and CVAE uses style features to constrain the hidden variable space, and finally generates text that retains the original semantics and conforms to the style of Yong Opera.

This model mainly generates colloquial dialogue lines and lyrics fragments that conform to the style of Yong Opera, which can be directly used for script creation and performance rehearsal. Enter the common sentences of modern Chinese, and the model can output text with corresponding semantics but the language style is converted into the characteristics of the Yongju dialect. Examples of specific input and output are as follows: enter “Don’t worry, sit down and talk slowly”, and the model output “Yimo is anxious, and sit down and talk slowly”; enter “The spring in Jiangnan is beautiful, and the peach blossoms are blooming”, and the model generates “The spring in Jiangnan is indeed commendable, and the pink peach blossoms are full of trees”.

While retaining the original meaning, the generated results incorporate dialect vocabulary unique to Yong Opera, such as “Yi” refers to “you”, “falling” means “coming down”, and local characteristic tone auxiliary words and opera rhythm are also added. With the help of style migration technology, the model not only completes the transformation of language and form, but also captures the unique expression and emotional colors of the original drama, so that the generated dialogue and lyrics are naturally smooth and have distinctive drama characteristics, which can be applied to the creation of new scripts and the digital reconstruction of traditional repertoire.

### Experiments

BiLingual Evaluation Understudy (BLEU) is an evaluation metric used to measure the degree of word overlap between the generated sequence and the reference sequence^30^. It adopts the “n-gram” matching mechanism, where n-gram refers to a phrase composed of n consecutive words. BLEU scores are usually converted to percentage notation. To calculate BLEU values, it is necessary to separately calculate the matching accuracy of each order of n-grams. Among them, the calculation method corresponding to n-gram is as follows.

6 $${Q}_{m}=\frac{\sum_{i}\sum_{j}min\left({f}_{j}\left({x}_{i}\right),{\begin{array}{c}max\\ k\in n\end{array}f}_{j}\left({h}_{ik}\right)\right)}{\sum_{i}\sum_{j}min\left({f}_{j}\left({x}_{i}\right)\right)}$$

In Eq. (6), ${max}_{k\in n}$ represents the number of times n-grams appear in multiple standard translations, while $min\left({f}_{j}\left({x}_{i}\right),{max_{k\in n}}\, {f}_{j}\left({h}_{ik}\right)\right)$ represents the minimum number of times n-grams appear in both standard and test translations. In the TFCPG model, the BLEU score is used to evaluate the quality of the generated text. It compares the text generated by the model with the real reference text manually marked to quantify the degree of matching between the two in the co-occurrence of n-element words. The test data is divided into two categories. One is the complete singing or dialogue fragments intercepted from the script of a classic musical as the real text, and the other is the candidate text generated by the model based on input conditions (such as style type, emotional label). The two need to correspond in semantics, sentence patterns and styles.

In order to ensure the scientific and fair comparison of baseline models (Transformer, PLM, GPT, BERT), all models are fine-tuned on the same data set. The data set studied in this paper is mainly collected and collated from Ningbo local libraries, local opera archives and other places. It contains about 120,000 words of classic Yong Opera scripts, lyrics manuscripts and other textual materials, as well as 230 paragraphs, a total of about 15 h of audio materials, and the text has been manually proofread and marked in dialects to ensure the unity and accuracy of the language style. In terms of model fine-tuning, each baseline model adopts a unified data preprocessing process: first, the script and lyrics are divided into words, and a vocabulary of 12,000 words constructed based on the Yongji dialect is used to remove duplicates, redundancy, and irrelevant characters; then the data is divided into training sets, verification sets, and test sets according to 8:1:1 to ensure that the style and content of each data set are consistent. Different models have their own parameter settings when fine-tuning. The Transformer model adopts a standard 6-layer encoder–decoder structure, the pre-training weights come from the WMT English–German translation task, and the Adam optimizer is used for fine-tuning. After 50 epoch training, the model with the highest BLEU verification set is selected for testing. PLM is based on HuggingFace’s GPT-2 medium-sized pre-training model, using the cross entropy loss function, after 30 epoch training, each round of evaluation verifies the degree of confusion and prevents overfitting.

The BERT model adopts the pre-training weights of BERT-base-Chinese and uses the Transformer-based decoder architecture. The training strategy is the same as PLM. In the evaluation phase, all models are carried out on the same test set, indicators such as BLEU-4 score and style conversion accuracy are calculated, and standard deviation and 95% confidence interval are reported. There are 500 test samples, which are manually screened by two Yong Opera experts to ensure that the language style is typical and the structure is complete. Through this series of unified and specific processes, it ensures that the performance comparison of different models has statistical significance and practical reference value.

**Fig. 3:**
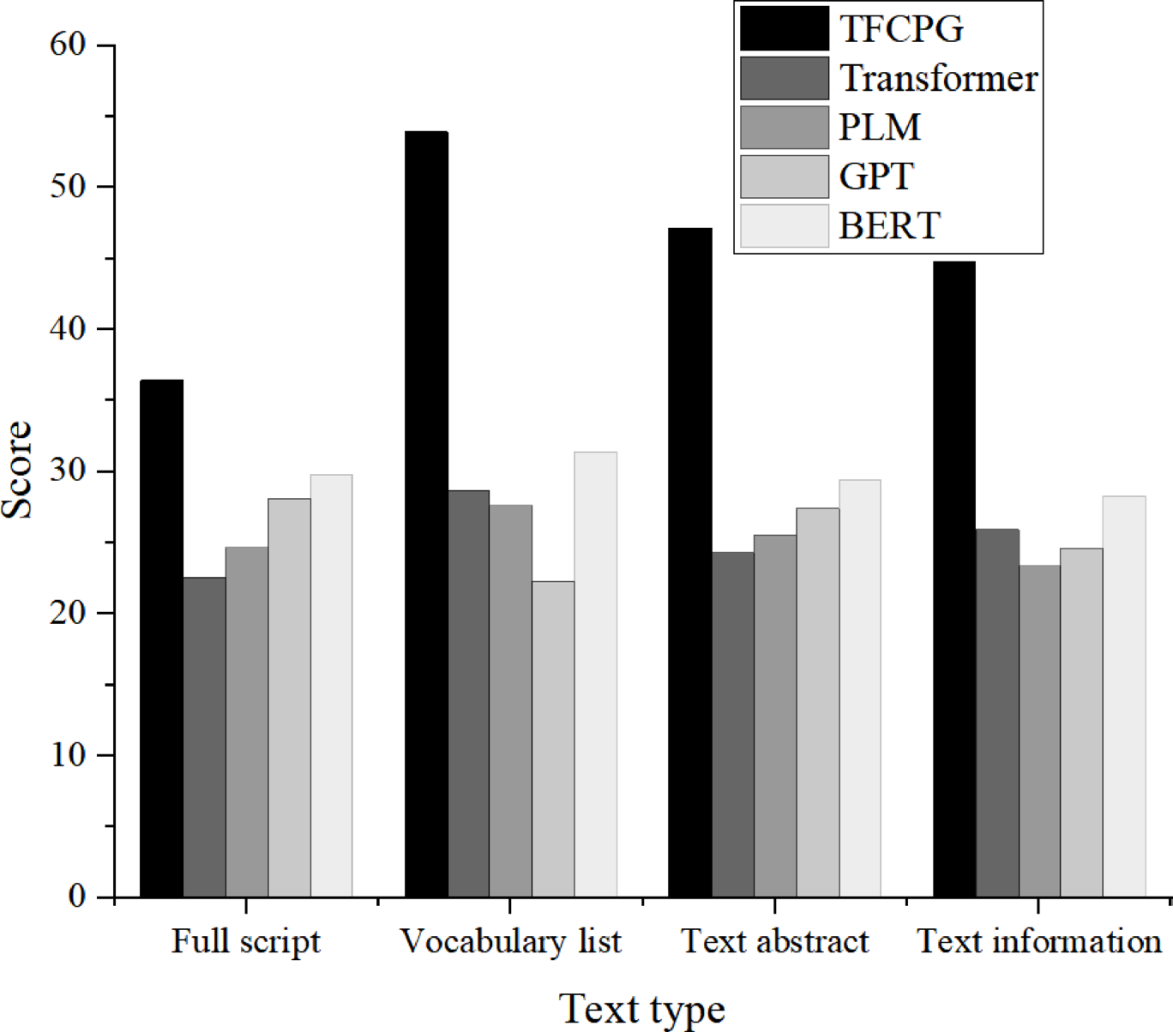
Comparison of BLEU scores generated by different text models.

In order to study the TFCPG model constructed in this article, the BLEU score is used to reflect the matching degree of automatically generated Yongju Opera language text. The research mainly focuses on the automatic generation of Yongju Opera text from four aspects: the full text of the script, vocabulary list, text abstract, and text information. The BLEU score obtained from the research is compared with the transformer model, pre-trained language model (PLM), GPT (generative pre-trained transformer), and BERT (bidirectional encoder representation from transformer) models. The specific comparison results are shown in Fig. 3.

As shown in Fig. 3, the TFCPG model is significantly superior to other models in many aspects. Specifically, in terms of the full script generation, TFCPG’s BLEU score is as high as 36.4 points, far exceeding the Transformer model’s 22.6 points, indicating that TFCPG is more outstanding in maintaining script coherence and style consistency. From the perspective of vocabulary list matching, TFCPG’s BLEU score is 53.9 points, which is 25.2 points, 26.3 points, 31.6 points, and 22.5 points higher than Transformer, PLM, GPT, and BERT, respectively. This demonstrates its outstanding ability in capturing and generating proprietary vocabulary for Yongju Opera. In contrast, other models score relatively low in these aspects. The average BLEU score of TFCPG on the four evaluation indicators is 45.55 points, far exceeding other models, and 20.175 points, 20.225 points, 19.95 points, and 15.825 points higher than the average scores of models constructed by Transformer, PLM, GPT, and BERT, respectively. These data fully demonstrate that the TFCPG model has significant efficiency and superiority in the automatic generation task of Yongju Opera language text. It not only performs well in overall script creation, but also demonstrates excellent performance in vocabulary selection, text summarization, and information transmission details. This provides important technical support for the digital protection and innovation of Yongju Opera culture.

The running speed of the model is one of the key indicators for evaluating system performance. In order to ensure that the TFCPG model can reach a convergence state within an acceptable time for users, detailed adjustments are made to the relevant parameters in the experiment. Meanwhile, user satisfaction is also a core indicator for measuring model performance. In order to comprehensively evaluate the performance of the model, three evaluation metrics are used, with scores automatically generated for the relevance, fluency, and consistency of the language text. The evaluation work is conducted by a panel of 10 experts in the field of Yongju Opera, and is scored on a 5-point scale. The text generated by the TFCPG model is utilized for comparative research with text generated based on Transformer, PLM, GPT, and BERT models. The comparison results are shown in Table 3.

**Table 3:** Performance comparison results of different text automatic generation models.

Model	Rise time	User satisfaction				BLEU
		Relevance	Fluency	Consistency	Overall satisfaction	
TFCPG	33.32 ± 2.15**	4.32	3.87	4.06	4.08	45.55 ± 1.32**
Transformer	76.81 ± 5.63	3.28	3.41	3.18	3.29	22.60 ± 2.41
PLM	64.83 ± 4.94	2.54	3.62	3.33	3.16	25.38 ± 1.89
GPT	55.26 ± 3.74	3.71	3.47	3.68	3.62	26.97 ± 2.18
BERT	58.94 ± 4.25	3.63	3.79	3.41	3.61	30.16 ± 1.95

*Indicates *p* < 0.05, **indicates *p* < 0.01 (compared to baseline models using two-sample t-test).

As shown in Table 3, the TFCPG model demonstrates significant advantages in runtime efficiency, with an average processing time of just 33.32 ± 2.15 min—significantly lower than all baseline models (*p* < 0.01). Compared to Transformer, PLM, GPT, and BERT models, TFCPG saves 43.49 min, 31.51 min, 21.94 min, and 25.62 min respectively. This statistically significant time efficiency greatly enhances user experience and practical application efficiency. In terms of user satisfaction, TFCPG excels across all key dimensions: correlation score of 4.32, fluency score of 3.87, consistency score of 4.06, and an overall satisfaction score of 4.08. The model achieves an outstanding BLEU score of 45.55 ± 1.32, a substantial improvement over traditional Transformer models (22.60 ± 2.41), with this advantage being highly statistically significant (*p* < 0.01). While Transformer, GPT, and BERT models perform well in general natural language processing tasks, none surpass TFCPG in text generation for the specific cultural context of Yongju opera. In summary, TFCPG’s exceptional performance in automatic language generation for Yongju not only validates its effectiveness and applicability in this cultural context but also paves a new technical pathway for the digital preservation and innovation of traditional opera culture.

In order to more comprehensively evaluate the performance of the model and verify the significance of the results, it is essential to introduce a statistical context. Providing standard deviations and confidence intervals (95% confidence intervals) for BLEU scores and other evaluation indicators can enhance the reliability of research conclusions. The details are shown in Table 4.

**Table 4:** Comparison of the performance of different models in the language generation task of Yongju.

Model	Standard deviation	Standard error	Sample size	*p* value
TFCPG vs. transformer	20.175	2.5	500	0.000***
TFCPG vs. PLM	20.225	2.6	500	0.000***
TFCPG vs. GPT	19.95	2.4	500	0.0008**
TFCPG vs. BERT	15.825	2.2	500	0.016**

*p < 0.1, **p < 0.05, ***p < 0.01.

As shown in Table 4, the BLEU score difference between the TFCPG model and the baseline model is highly significant. All comparison *p* values are less than 0.05. When compared with Transformer, PLM, and GPT, the *p* value is 0.000. Its advantages are extremely outstanding, and the possibility of random errors is basically excluded. Compared with these three models, TFCPG has an average improvement of 20.175 points, 20.225 points, and 19.95 points, respectively, with a standard error of 2.4–2.6, indicating that under the same test conditions, the quality of the generated text is significantly higher, and the script performs well in terms of coherence and style unity. The difference between TFCPG and the BERT model is slightly smaller, but the *p* value is 0.016, which is statistically significant, with a standard error of 2.2, and the results are highly credible.

Ablation Study (Ablation Study) is an important method to evaluate the contribution of each component in the model to the overall performance. By systematically removing or modifying certain parts of the model, the unique value of each component and its impact on the final result can be better understood. In this study, in order to fully demonstrate the effectiveness of the TFCPG and CVAE integrated models, especially the individual effects of key components such as keyword extraction and multitasking learning, detailed ablation experiments were conducted. The details are shown in Table 5.

**Table 5:** Ablation experiment.

Model configuration	BLEU score average	Style conversion accuracy	Cosine similarity	Confusion	User satisfaction
Complete TFCPG + CVAE model	45.55	0.9428	0.9182	102.34	4.32
TFCPG model without keyword extraction	40.55	–	–	–	3.89
TFCPG model without MTL (script generation only)	31.5	–	–	–	3.71
CVAE model of styleless mapping network	38.9	0.8736	0.8512	110.45	4.02

Table 5 presents the results of the ablation study, highlighting the importance of the complete TFCPG + CVAE model and its components. The complete model integrates a variety of optimization techniques and has the best performance. Its BLEU score has an average of 45.55, style conversion accuracy is 0.9428, cosine similarity is 0.9182, confusion is 102.34, and user satisfaction score is 4.32. The model makes full use of the advantages of Transformer architecture, combines keyword extraction, word segmentation technology, and multitasking learning strategies to improve the quality of generated text; the CVAE style mapping network enables the generated content to conform to the norms of classical drama, which is both practical and innovative, proving that this comprehensive framework has obvious advantages in language generation and style migration tasks of specific cultural backgrounds. The performance of the TFCPG model without keyword extraction decreased significantly, the average BLEU score dropped to 40.55, and the user satisfaction score dropped to 3.89. This shows that keyword extraction has a significant role. As the core vocabulary of the original text, it provides constraints for generation to ensure consistency with the original script. After removal, the model’s ability to capture and express key information is weakened, affecting the overall quality. The average BLEU score of the TFCPG model without MTL (script generation only) was only 31.5, and the user satisfaction score dropped to 3.71. Multitasking learning optimizes performance by processing multiple related tasks at the same time.

Although the input is the same, the output goals are different. Experiments have shown that MTL improves the performance of individual tasks, enhances the generalization ability of the model, and makes language expression smoother and more natural. For the CVAE model without a style mapping network, the consistency of the generated text style and the conversion accuracy rate decreased, the average BLEU score decreased to 38.9, the style conversion accuracy rate decreased to 0.8736, etc., and the user satisfaction score decreased to 4.02. The style mapping network can mine semantic information and transform it into style feature vectors to ensure that the generated content conforms to the target style. In summary, the ablation research shows in detail the importance of components such as keyword extraction, multitasking learning, and style mapping network in the integrated model, verifies its positive contribution to model performance, and provides a reference for future research. It helps to deeply understand the working mechanism of the model and lays the foundation for further optimization and improvement.

In the Yongju language generation and migration model constructed in this paper, “Modern language semantics” is widely used. The word embedding layer adopts advanced representation learning methods to convert text into high-dimensional vectors, consider the basic meaning and context of vocabulary, and help the model capture the unique semantics of Yongji dialect and ancient vocabulary to ensure that the generated text is faithful and ideologically correct. Transformer-based TFCPG introduces a multi-head attention mechanism, allowing the model to consider the entire input text semantics when generating new vocabulary, and capture long-distance dependencies. The multi-task learning strategy is adopted, the two-way encoder–decoder structure is integrated, and the self-regression and self-coding pre-training are combined to help the model understand the deep cultural background and emotional color of the text, and express the artistic characteristics of Yong Opera. However, it is still a difficult problem to realize the combination of modern language semantics and opera style, and to accurately quantify the integration into the artistic expression of Chinese opera. The research focuses on the establishment of a style library, and quantifies the key elements as style feature vectors for model learning. Although it enhances expressiveness, it requires a large amount of high-quality data and is limited by the capabilities of existing algorithms.

## Construction of language style mapping model for Yongju opera

The task of text style transfer is to change the expression of the original text by applying target style information while maintaining its original meaning^31,32^. If sentence sets of different style types are defined as two hypothesis spaces, $A=\left\{{a}_{1},{a}_{2},\ldots ,{a}_{m}\right\}$ and $B=\left\{{b}_{1},{b}_{2},\ldots ,{b}_{m}\right\}$ respectively, then the task of the style transfer model is to map any sample in one space *A* to another space *B*, and this mapping is bidirectional. The details are shown in Eqs. (7) and (8).

7 $${B}^{\prime}={h}_{a\to b}\left(A,{f}_{b}\right)$$

8 $${A}^{\prime}={h}_{b\to a}\left(B,{f}_{a}\right)$$

**Fig. 4:**
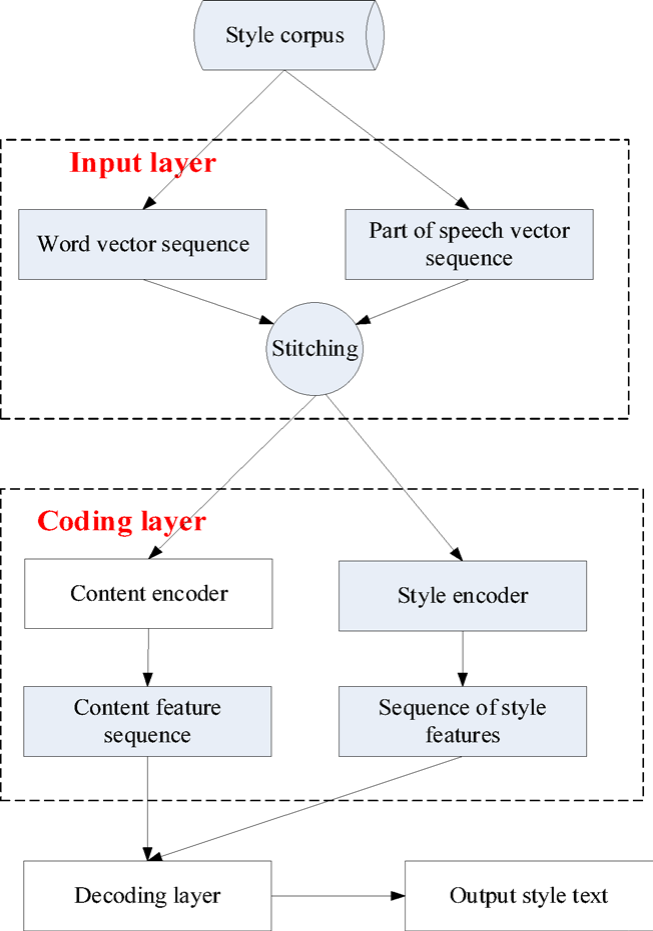
Overall framework diagram of the model.

Currently, research on text style transfer mainly focuses on emotional style, situational style, and gendered style, and most of the studies are conducted in the English field, with relatively less research in the Chinese field. In addition, due to the difficulty of obtaining parallel data, traditional deep learning models face challenges in distinguishing content and style, making it difficult to learn content and style features that correspond to specific semantic information. To address these issues, this article proposes a text style transfer mapping model based on conditional variational autoencoders (CVAE). In the context of the TFCPG model for automatically generating Yongju Opera language text, the main goal of this style mapping model is to convert the generated Yongju Opera language according to the set style requirements to meet specific artistic, cultural, or emotional needs, thereby enhancing the expressiveness and viewing value of the text. The model architecture studied in this article is shown in Fig. 4.

As shown in Fig. 4, the model studied in this article includes a style corpus, an input layer, an encoding layer, and a decoding layer. During the operation of the model, the input text is first transformed into a representation matrix containing word meanings and parts of speech through embedding techniques. Next, two bidirectional gate recurrent units (GRUs) are used to extract content information and style information from the text. According to actual needs, the layer is adjusted to modify the extracted style information accordingly. Finally, the decoding layer converts the processed information into target style text output.

When building this model, special attention needs to be paid to establishing a style library. Yongju Opera, as a local drama with diverse language styles, requires specific language styles to present its performance scenes, emotional expressions, and character settings. In order to accurately capture these style features, key elements such as phonology, sentence structure, vocabulary selection, and emotional color in the text data of Yongju Opera are analyzed in depth and quantified into specific style feature vectors. By using style mapping algorithm to learn and train a large number of Yongju Opera texts, a feature vector set matching various styles is generated, providing strong support for subsequent style transformation. In addition, various preprocessing measures are taken in the style corpus processing stage, and the entire corpus is transformed into a graph structure form that is easy for the model to process. Assuming a certain sentence in corpus D is $M=\left\{{m}_{1},{m}_{2},\ldots ,{m}_{K}\right\}$, where *m* is a preprocessed word, and the set of style labels in corpus D is $N=\left\{{n}_{1}, {n}_{2}\right\}$. The details are shown in Eq. (9).

9 $$H=\left\{connect\left({m}_{i},{n}_{j}\right)|{m}_{i}\in M,{n}_{j}\in N\right\}$$

This article uses the CVAE model to deeply explore the semantic information of text. The CVAE model consists of two main parts: an encoder and a decoder. Among them, the encoder is responsible for learning and extracting the features of the input text, compressing these features into a low dimensional and informative vector. The decoder uses this compressed vector to restore the original input text as accurately as possible. Compared with existing text style transfer methods, the model proposed in this article has unique features. Two text sequences are processed simultaneously and labeled as text ${A}_{1}={\left\{{A}_{1}^{k}\right\}}_{k=1}^{n}$ and text ${A}_{2}={\left\{{A}_{2}^{k}\right\}}_{k=1}^{n}$, respectively. *n* is the number of text pairs; ${A}_{1}^{k}$ represents the first text sequence of the k-th input text pair; ${A}_{2}^{k}$ represents the second text sequence of the k-th input text pair. In order to better capture the semantic essence of input text and generate high-quality target text, the GRU model is chosen to construct the encoder and decoder. During the encoding process, the GRU model combines the hidden state of each word from the previous moment with the information of the current word, and precisely regulates the information flow in the network through its sophisticated gating structure. In this way, key information can be effectively compressed and stored in the hidden state f. In the decoding stage, the decoder predicts the next word based on the hidden state f and all previously generated words. The probability of each word being generated is represented as t, and high-precision reconstruction of the input text is achieved by maximizing this probability. This design not only enhances the semantic understanding ability of the model, but also lays a solid foundation for its outstanding performance in text generation tasks.

The core component of the CVAE-based text style transfer mapping model is the style mapping network. The main task of this network is to accurately map the language content of Yongju Opera into a specific style space. To train this network, a deep learning model is employed and a multi-head attention (MHA) framework is integrated to efficiently encode the input target language text variants^33^. The construction of this network is quite sophisticated, including convolutional layers, seven convolutional blocks, and activation functions. Here, each convolutional layer uses a 4 × 4 convolution kernel and downsamples with a stride of 2. In order to capture the Yongju Opera language style features output by each activation function layer, these feature maps are read line by line, and the maximum value is selected to represent the corresponding target style features. At the same time, in order to ensure that these style features match the language features in the language text style transfer network, padding and concatenation methods are used for summarization. At the same time, by using a 1 × 1 convolution kernel, it is possible to flexibly control the depth of style features and adjust the linear combination of information in the channel dimension. This not only achieves consistency between style features and language features in terms of the number of channels, but also promotes the integration of information exchange between ascending and descending dimensions and across channels. Finally, these carefully processed style features are fed into the transposed convolution block of the Yongju opera language text style transfer network to achieve language style mapping.

The main function of the decoding layer is to restore the encoded feature vectors to text. During the decoding process, the hidden state is decomposed into two parts: content features and style features. Content features are mainly responsible for constructing the main structure of sentences, while style features are responsible for making detailed adjustments to the generated results. To achieve this goal, different methods are used to decode these two features, and the decoding results are ultimately merged to output the probability of each vocabulary. The decoding of text features is accomplished through a GRU model. The calculation formulas for the output vocabulary ${\widehat{m}}_{f}^{\left(k\right)}$ of the model at time *k* are as follows:

10 $${s}^{\left(k\right)}=GRU\left({f}^{\left(k\right)},\left[{\widehat{a}}^{\left(k-1\right)}\right]\right)$$

11 $${\widehat{m}}_{f}^{\left(k\right)}=softmax\left(\sigma \left({M}_{of}{s}^{\left(k\right)}+{y}_{of}\right)/\beta \right)$$

In Eqs. (10) and (11), ${\widehat{a}}^{\left(k-1\right)}$ is the word vector of the output vocabulary from the previous moment; $s\left(k\right)$ is the output vector of the decoder; ${\widehat{m}}_{f}^{\left(k\right)}$ is the probability of words based solely on content vector output; $\beta$ is the temperature coefficient used to adjust the smoothness of probabilities during training.

Style features play a fine-tuning role in text generation to prevent output text from overly relying on specific styles. Therefore, a simple method is adopted to handle the decoding of style features: through a layer of feedforward network, the style features are mapped to vectors of the same length as the word list, and then the softmax function is applied to convert them into probability distributions. As shown in Eq. (12):

12 $${\widehat{m}}_{h}^{\left(k\right)}=softmax\left(\sigma \left({M}_{oh}{s}^{\left(k\right)}+{y}_{oh}\right)/\beta \right)$$

The final output word probability is the weighted average of the content vector and style feature output probabilities. To ensure the dominant position of content in the generated text, the output weight of the content vector is set to be higher than the output weight of the style feature. The specific weight values are learned through training data, which can flexibly incorporate the required style elements while maintaining the coherence of the text content.

## Experiments of Yongju opera language generation transfer model

The code writing and training in this article are based on the Pytorch deep learning framework, and the basic parameter settings are shown in Table 6.

**Table 6:** Training parameters.

Serial number	Parameter name	Set value
1	Word vector length (vocabulary)	128
2	Word vector length (part of speech)	128
3	Hidden space size (GRU)	256
4	Temperature coefficient	100
5	Batch size	32
6	Optimizer	Adam
7	Preliminary test learning rate	0.01
8	Calculation framework	Tensorflow 1.4
9	Running memory	32 Gigabyte

As shown in Table 6, regarding the setting of model parameters, the sizes of word vectors and part of speech vectors are both set to 128, while the hidden space size of GRU is set to 256, and both adopt a single-layer structure. During the training process, the initial temperature coefficient is set to 100, and the default values of Pytorch are used for other parameters.

The core goal of text style transfer task is to achieve the transformation of text style while preserving the information of text content. In order to comprehensively evaluate the performance of the model, this article mainly considers four key indicators. Firstly, regarding style transfer accuracy, the higher the accuracy, the closer the style of the generated text is to the target style, reflecting the strong ability of the model in style transfer. Secondly, the evaluation of content retention relies on cosine similarity, where a higher cosine similarity value indicates that the generated text is closer in content to the original text. Furthermore, the fluency and grammatical compliance of the generated text are also key factors in evaluating the performance of the model. The fluency of the text is mainly quantified through the indicator of perplexity. The lower the perplexity value, the more grammatically correct the generated sentences, indicating that the model has strong ability in generating high-quality sentences. Finally, there is the BLEU score.

To comprehensively evaluate the stability of model performance and eliminate random factors, this study systematically implemented multiple uncertainty control mechanisms. During the experimental design phase, all comparative experiments conducted 10 independent replicates, with model performance variability assessed through mean value and standard deviation calculations. To address dataset bias, stratified sampling was employed to construct training and test sets, ensuring balanced distribution of Yongju opera texts across different historical periods and styles. To control randomization effects, all neural network models utilized fixed random seeds and were trained/inferred under identical hardware conditions. Experimental results demonstrated that TFCPG model metrics showed significantly lower standard deviations than baseline models across repeated trials, confirming statistically consistent and reproducible performance improvements. These comprehensive uncertainty control measures collectively ensured the reliability and scientific validity of reported performance enhancements.

**Fig. 5:**
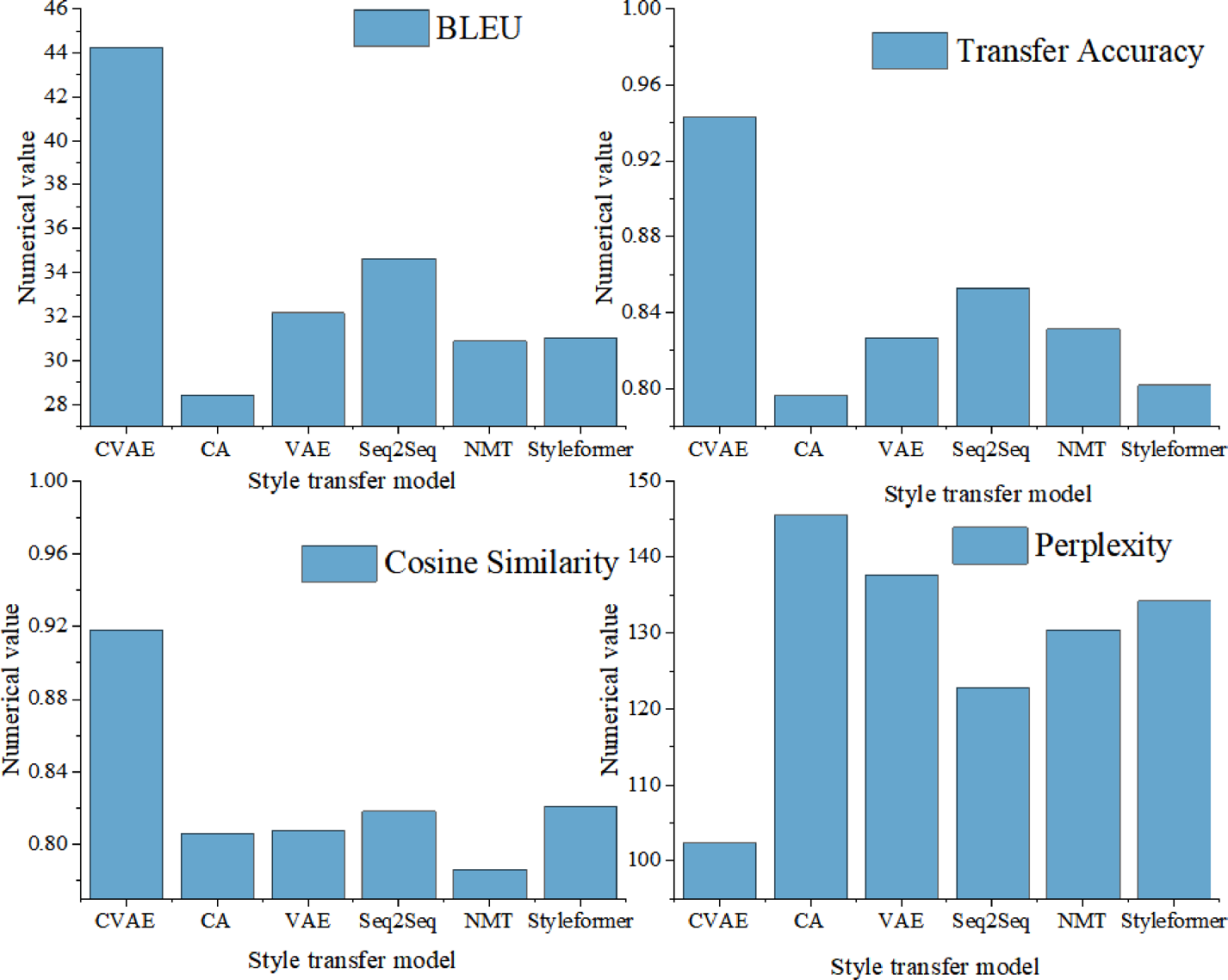
Performance comparison of text style transfer mapping models in different languages.

To verify the effectiveness of the research method in this article, a dataset is constructed using the data collected in Table 2. The data collected in Table 2 mainly includes five types: script full text, audio files, vocabulary list, text abstract, and text information. By extracting content features such as phonology, sentence structure, vocabulary, and emotional color from these data types, the language style transfer mapping of Yongju opera is completed. To verify the performance of the CVAE-based language text style transfer mapping model, a comparative study is conducted on the language text style transfer mapping models constructed in this article, including the cross alignment (CA) model, variational auto-encoders (VAE), sequence to sequence (Seq2Seq) model, neural machine translation (NMT), and Styleformer model. The specific comparison results are shown in Fig. 5.

As shown in Fig. 5, the CVAE model exhibits significant advantages in all evaluations, with a BLEU score of up to 44.26, indicating that the generated text is highly similar to the target style text. At the same time, the accuracy of style transfer reaches 0.9428, indicating that the style of the generated text is very close to the target Yongju opera style, demonstrating the powerful style transfer ability of the model. In addition, the cosine similarity is 0.9182, reflecting that the model effectively preserves the original content during style transfer, avoiding information loss. In terms of text fluency, the perplexity level is as low as 102.34, proving that the generated sentence structure is smooth, grammatically correct, and naturally fluent. Compared with other models, the comprehensive performance of the CVAE model is superior. The CA model performs poorly in BLEU score and style transfer accuracy, with scores of 28.43 and 0.7963, respectively, indicating significant shortcomings in aligning with the target style and preserving the original content. However, models such as VAE, Seq2Seq, NMT, and Styleformer, although each has its own characteristics, have not surpassed the CVAE model in overall performance. In summary, the CVAE-based Yongju Opera language text style transfer mapping model studied in this article has shown excellent performance in multiple key indicators, bringing new breakthroughs and innovative methods to the study of text style transfer.

**Fig. 6:**
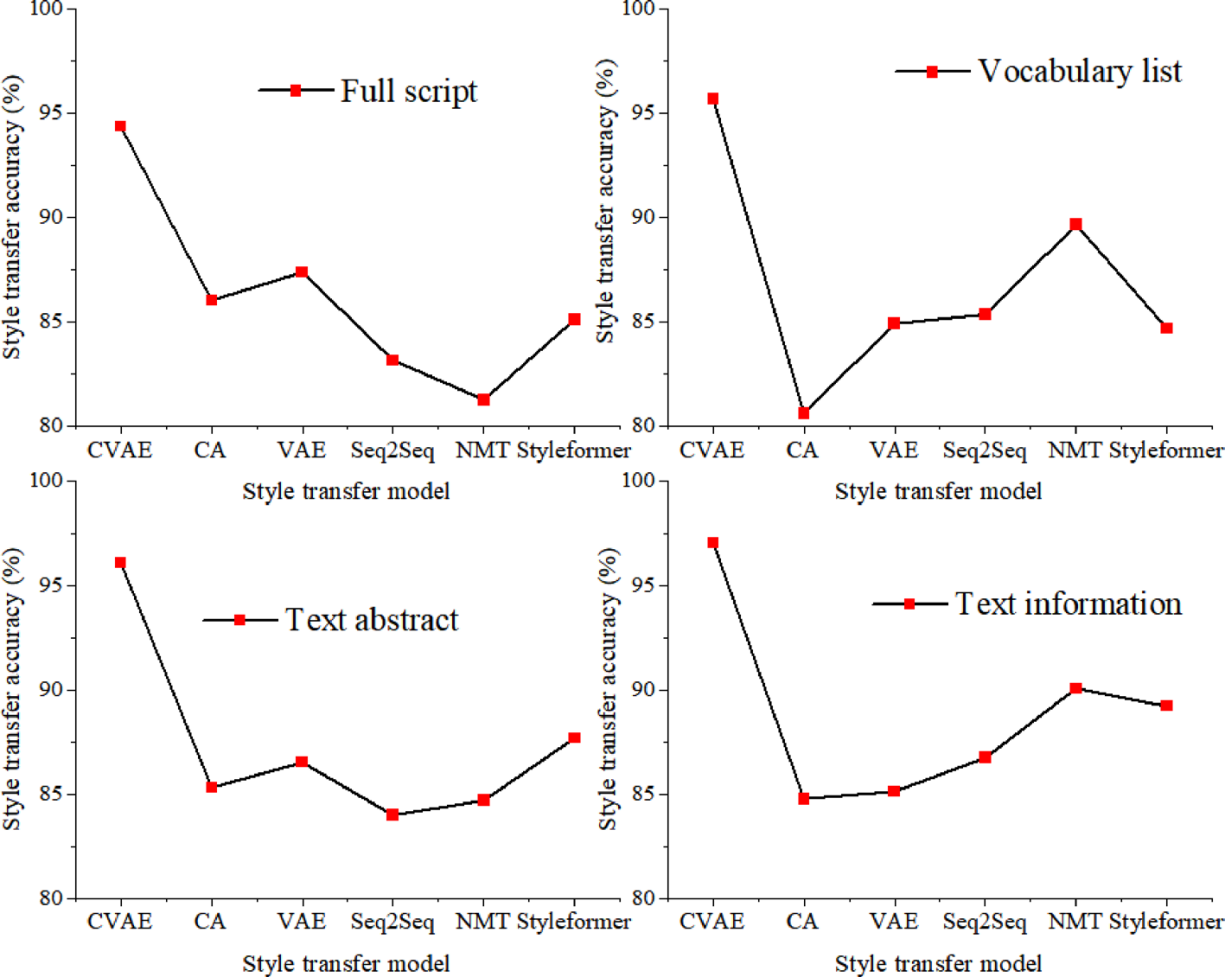
Comparison of language text style transfer mapping models for language text transfer accuracy.

The data collected in Table 2 mainly includes four types: full script, vocabulary list, text abstract, and text information. Four types of data are studied, and the CVAE language text style transfer mapping model constructed in this article is utilized to perform style transfer mapping on these four types of text data. Firstly, the core capability of the model is tested by quantitatively evaluating the consistency between the transferred text and the target style, measuring the model’s ability to successfully transform the text style while maintaining the content unchanged, thereby obtaining the accuracy of style transfer. The accuracy of style transfer obtained is compared with models constructed based on CA, VAE, Seq2Seq, NMT, and Styleformer. The specific comparison results are shown in Fig. 6.

As shown in Fig. 6, this article uses CVAE to construct a text style transfer mapping model, and conducts in-depth style transfer experiments on four categories of text data: full script, vocabulary list, text abstract, and text information using this model. The experimental results show that the CVAE model performs excellently in style transfer of various types of text data, and can effectively transform text styles while maintaining the integrity of the original content, achieving high accuracy in style transfer. Especially in the style transfer of text information, the CVAE model significantly outperforms other comparison models with an accuracy of 97.03%, which is 12.25%, 11.91%, 10.28%, 6.97%, and 7.82% higher than the models constructed by CA, VAE, Seq2Seq, NMT, and Styleformer, respectively, highlighting its unique advantages in handling complex text structures and emotional expressions. In addition, in the style transfer experiments of vocabulary lists and text summaries, the CVAE model also achieves high accuracies of 95.67% and 96.08%, respectively, fully verifying the model’s wide adaptability to different types of text data. The average accuracy of the research method for style transfer mapping in these four types of text is 95.79%, which is 11.6%, 9.8%, 10.97%, 9.36%, and 9.11% higher than the models constructed by CA, VAE, Seq2Seq, NMT, and Styleformer, respectively. By quantitatively evaluating the consistency between the transferred text and the target style, this article successfully verifies the core advantage of the CVAE model in text style transfer tasks, which is the ability to accurately transform text styles while maintaining content consistency, thus providing solid support for research in the field of text style transfer.

## Conclusions

As a traditional opera in Ningbo, Zhejiang, Yong Opera carries deep historical and regional cultural connotations with its unique singing, performing arts and rich repertoire. However, under the impact of modern fast-paced life, traditional opera faces dilemmas such as audience loss and difficult inheritance. In order to help the modernization and development of Yong Opera, this research uses AIGC technology to explore the automatic generation and style migration of Yong Opera language. The research first analyzes the uniqueness of the Yong Opera language in depth, and carefully constructs a rich data set containing classic and modern scripts, singing audio, and dialect vocabulary to build a solid foundation for model training. In terms of model selection, the TFCPG model, with its excellent generation ability and efficiency, has become the best choice for automatic generation of Yongqu language text; the CVAE model performs well in style migration, and can accurately integrate modern language semantics with Yongqu style, so that the generated content not only retains the artistic characteristics of Yongqu, but also conveys the core meaning of the original text. This study confirms the potential of AIGC technology in the field of opera language generation and migration, infuses new impetus into the inheritance and innovation of Yong Opera culture, reproduces the charm of Yong Opera language through digital means, and opens up a new path for the protection and inheritance of traditional opera culture.

## Data Availability

The data that support the findings of this study are available on request from the corresponding author upon reasonable request.

## Code availability

The source code used in this study is publicly available at: [URL to Zenodo]. 10.5281/zenodo.17725656.
